# Interaction of genotype-ecological type-plant spacing configuration in sorghum [*Sorghum bicolor* (L.) Moench] in China

**DOI:** 10.3389/fpls.2022.1076854

**Published:** 2023-01-12

**Authors:** Peng Yan, Ying-Hui Song, Kuang-Ye Zhang, Feng Zhang, Yu-Jie Tang, Xiang-Na Zhao, Nai Wang, Fu-Lai Ke, Feng-Ju Gao, Ji-Hong Li, Jun-Xia Li, Yue Gao, Wei Yang, Fang-Chao Gao, Dan-Dan Qi, Zhi Wang, Guang-Xia You, Fen-Xia Han, Zi-Yang Zhou, Gui-Ying Li

**Affiliations:** ^1^ Institute of Crop Sciences, Chinese Academy of Agricultural Sciences, Beijing, China; ^2^ Cereal Crop Institute, Henan Academy of Agricultural Sciences, Zhengzhou, Henan, China; ^3^ Sorghum Research Institute, Liaoning Academy of Agricultural Sciences, Shenyang, Liaoning, China; ^4^ Bureau of Agriculture and Rural Affairs of Xinle City, Xinle, Hebei, China; ^5^ Crop Resources Institute, Jilin Academy of Agricultural Sciences, Gongzhuling, Jilin, China; ^6^ Xingtai Academy of Agricultural Sciences, Xingtai, Hebei, China; ^7^ Dezhou Academy of Agricultural Sciences, Dezhou, Shandong, China

**Keywords:** sorghum, genotype-environment-management interaction, plant space configuration, variety introduction, ecological type

## Abstract

Grain sorghum has been a significant contributor to global food security since the prehistoric period and may contribute even more to the security of both food and energy in the future. Globally, precise management techniques are crucial for increasing grain sorghum productivity. In China, with diverse ecological types, variety introduction occasionally occurs across ecological zones. However, few information is available on the effect of ecological type on genotype performance and how plant spacing configuration influences grain yield in various ecological zones. Hence, a series of two-year field experiments were conducted in 2020 and 2021 in four ecological zones of China, from the northeast to the southwest. The experiments included six widely adapted sorghum varieties under six plant spacing configurations (two row spacing modes: equidistant row spacing (60 cm) mode and wide (80 cm)-narrow (40 cm) row spacing mode; three in-row plant spacings: 10 cm, 15 cm, and 20 cm). Our results indicated that ecological type, variety, and plant spacing configuration had a significant effect on sorghum yield. Ecological type contributed the highest proportion to the yield variance (49.8%), followed by variety (8.3%), while plant spacing configuration contributed 1.8%. Sorghum growth duration was highly influenced by the ecological type, accounting for 87.2% of its total variance, whereas plant height was mainly affected by genotype, which contributed 81.6% of the total variance. All test varieties, developed in the south or north, can reach maturity within 94-108 d, just before fall sowing in central China. Generally, sorghum growth duration becomes longer when a variety is introduced from south to north. A late-maturing variety, developed in the spring sowing and late-maturing regions, possibly could not reach maturity in the early-maturing region. The row spacing modes had no significant affect on sorghum yield, but the equal-row spacing mode consistently caused higher yields with only one exception; this might imply that equal-row spacing mode was more advantageous for boosting sorghum yield potential. In contrast, decreasing in-row plant spacing showed significant positive linear associations with sorghum grain yield in most cases. In addition, these results demonstrated that sorghum is a widely adapted crop and enables success in variety introduction across ecological zones.

## 1 Introduction

Sorghum [*Sorghum bicolor* (L.) Moench] is the world’s fifth most important cereal crop with a global production of over 62 million tons in 2020 (USDA, 2021). The inherent adaptation of sorghum to marginal lands and, more importantly, the ability to produce a high yield have both made it a dietary staple for millions of people living in the subtropical and semi-arid regions of Africa and Asia (Hariprasanna and Rakshit, 2016). Sorghum cultivation has a long history in China, and modern sorghum farming began in the early 20^th^ century (Gao et al., 2010). In 2020, China produced over 3.6 million tons of sorghum. However, during the past few years, China has been forced to import over 5 million tons of sorghum grain annually due to the restricted supply (Wang and Zou, 2020). Therefore, enhancing grain sorghum production is necessary in such a context. Additionally, a better understanding of how sorghum responds to various cultivation management practices would enable the narrowing of the sorghum yield gap and ensure food security, particularly in arid and semi-arid regions. China is a large country with a myriad of ecological environments. Scant research exists on the interaction of genotype, cross-ecological environment, and cultural practices.

**Figure 1:**
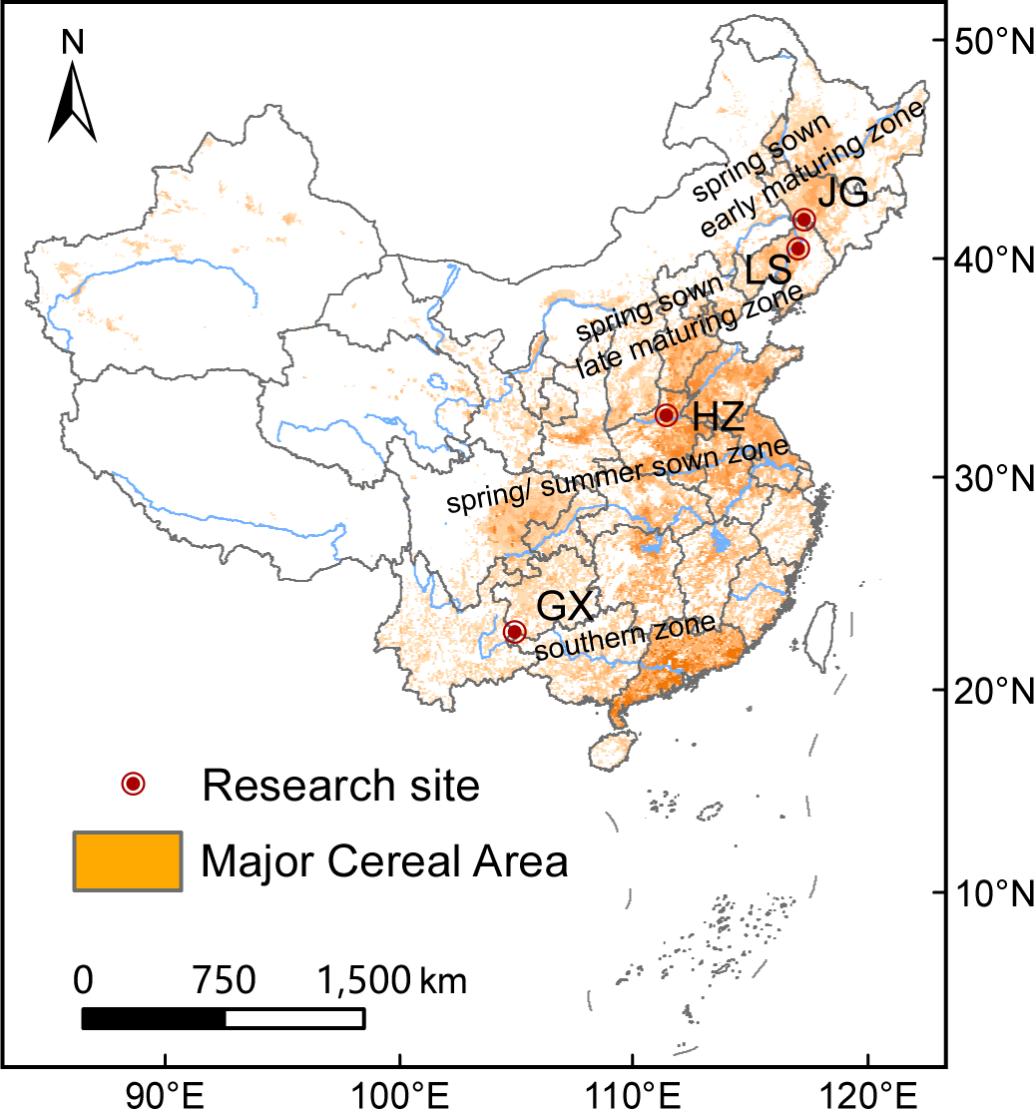
Four mainly sorghum ecological zones in China and the site of experimental sites at each zone: Gongzhuling in Jilin province (JG), Shenyang in Liaoning province (LS), Zhengzhou in Henan province (HZ), and Xingyi in Guizhou province (GX).

In the past four decades, sorghum grain yield in the top ten sorghum-producing countries in the world increased annually at 0.96% yr^-1^, and China has experienced a phenomenal yield gain, with an increase of roughly 100.9 kg ha^-1^ yr^-1^ (Rakshit et al., 2014). Currently, the majority of China’s sorghum production is centered in four ecological zones, according to varieties planted, climatic conditions, soil types, and cultivation systems (Diao, 2017). These are known as the spring-sown early-maturing zone, the spring-sown late-maturing zone, the spring/summer-sown zone, and the southern zone (**Figure 1**); they made up roughly 45.0%, 40.0%, 13.0%, and 2.0% of the total sorghum area in China, respectively (Gao et al., 2010). In the spring-sown early and late-maturing zones, the total effective cumulative daily average temperature (≥ 10°C) reached 2000-3000°C and 3000-4000°C, respectively; annual rainfall ranged from 100-700 mm and 16.2-900 mm, respectively. The major constraint to sorghum production was drought, especially in the early sorghum growing stage (Wang et al., 2007). The summer zone and the southern zone have high annual rainfall (≥ 600 mm) and abundant heat and light (Jiang et al., 2019; Cao et al., 2020). However, sorghum production is constrained by the fact that it is typically grown in lowlands that are flooded with water or dry places, or hillsides in these two zones.

Genotype selection is essential to enhance sorghum productivity. In China, sorghum breeding began in the mid-1950s, and significant progress has been achieved during the past 60 years (Li and Li, 1998; Gao et al., 2010). However, sorghum yield continues to lag behind other crops such as maize, rice, and wheat. A principal target of crop breeders is to improve the yield potential, which is connected to on-farm production. Genetic advancements related to sorghum breeding have slowed in recent years, which has resulted in a lower yield potential than that of other cereals (Rakshit et al., 2014; Xu et al., 2017). Moreover, the extensive use of foreign germplasm has led to the decline of the broad adaptation of Chinese sorghum, especially in the northern low-latitude regions, and caused slow germination and juvenile development. It has even caused a yield penalty (Gao et al., 2010). Sorghum varieties with broad adaptation can produce stable yields across a variety of growing conditions; site-specific information on variety performance is critical for improving sorghum yield in diverse ecological conditions. A better understanding of the complexity of genotypic fit for the environment and management practices could help maximize sorghum yield potential in China.

Sorghum grain yields were considerably influenced by in-row plant spacing (plant density), and field crops such as maize exhibited a trend toward higher yields as plant density increased (Liu et al., 2021). However, sorghum could produce tillers, especially under low plant density, which could minimize the impact of plant density on grain yield. Conley et al. (2005) indicated a sorghum yield-plant density linear-plateau response in Missouri, where sorghum yield increased from 6.3 Mg ha^-1^ to 7.3 Mg ha^-1^ when plant density increased from 73,600 to 147,300 plants ha^-1^; however, yield benefits plateaued at 368,000 plants ha^-1^. Later in Kansas, Pidaran (2012) found a positive yield response (14.0%) when sorghum plant density was increased from 24,000 to 96,000 plants ha^-1^ in some locations, but no positive yield response was found in others. Presently, the plant density of grain sorghum in the spring-sown early-maturing zone is between 200,000 and 300,000 plants ha^-1^, which is the largest sorghum plant density in China. Moreover, Yang et al. (2021) showed that a planting density of 300,000 plants ha^-1^ could constitute a suitable production density to balance the individual plant photosynthetic level and sorghum yield in this region. Nevertheless, the optimum plant density depended largely on sorghum variety, growing conditions, and management practices. Blum (1970) found that the yield of the late-maturing sorghum variety was the highest under low plant density, while the yield of the early-maturing sorghum variety was the highest under high density. Berenguer and Faci (2001) indicated that a high plant density did not present a productive advantage in the sorghum grain yield, especially under limited irrigation conditions. However, previous study showed significant interaction of plant density, nitrogen rate, and variety on sorghum grian yield as well (Dembele et al., 2021). Therefore, plant density in terms of grain yield varied with different growing conditions and sorghum varieties; therefore, knowledge of optimum plant density in combination with cultivation conditions and variety will help producers boost sorghum productivity.

Reducing row spacing has been considered as an efficient management strategy to promote crop canopy closure, lower evaporation, and increase evapotranspiration efficiency. In this case, yield response to narrow rows is directly associated with an improvement in light interception early in the season under non-stress conditions. Staggenborg et al. (1999) indicated that grain sorghum yield increased by 10.0% when the row spacing decreased from 75 cm to 25 cm in a high-yield environment. Maiga (2012) showed that sorghum yield increased by 3%-14% with narrow rows (25 cm) compared with wide row spacing (75 cm) in different test environments during the same season. A review of research from the 1980s to 2011 revealed that narrow row spacing increased sorghum yield by 0.5 mg ha^-1^ in more than 75% of all observations when compared to wide row spacing. Moreover, sorghum yield increases were consistent when sorghum yield was above 6.0 Mg ha^-1^. Nevertheless, under lower-yield conditions, narrow rows slightly promoted sorghum grain yield in comparison with wide rows. Fernandez et al. (2012) documented that wide row spacing (76 cm) plantings at 170,000-240,000 plants ha^-1^ produced a higher grain yield than the narrow row spacing (38 cm) at the same plant density. Thus, similarly to plant density, the impact of row spacing on sorghum grain yield varied across the sorghum variety, growing environment, and management factors, and there is a lack of clear consensus on which row spacing is more appropriate.

Hence, in the current study, we explore the interaction of sorghum genotype, ecological type, and plant spacing configuration at the four major grain sorghum production areas in China. The specific objectives of this study were to: (1) quantify grain sorghum response to ecological type, genotype, plant spacing (including plant density and row spacing modes), and their interactions; and (2) optimize variety and plant spacing for the four major sorghum production areas in China to boost sorghum productivity.

## 2 Material and methods

### 2.1 Materials and field experimental design

Field experiments were conducted in 2020 and 2021, and four sites were selected to represent four ecological zones of sorghum production (**Figure 1**): Gongzhuling city in Jilin province (JG) representing the spring-sown early-maturing zone; Shenyang city in Liaoning province (LS) representing the spring-sown late-maturing zone; Zhengzhou city in Henan province (HZ) representing the summer-sown zones; and Xingyi city in Guizhou province (GX) representing the southern zone. The cropping systems were a spring sorghum monocropping system in JG and LS and a winter wheat-summer sorghum double cropping system in HZ and GX. The initial statute of the topsoil layer (0-20 cm) at the four experimental sites is shown in **Supplementary Table S1**.

**Figure 2:**
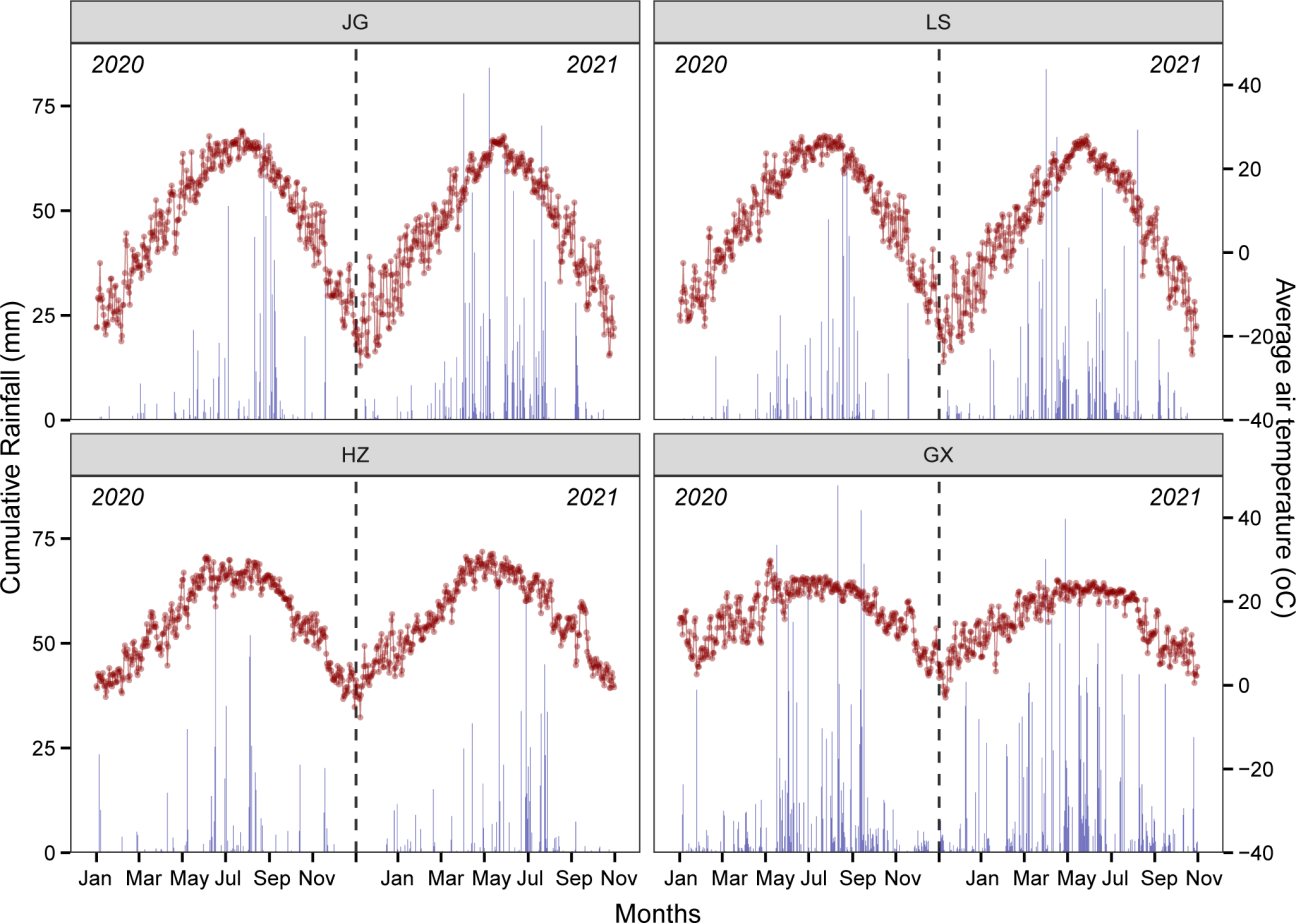
Weather conditions including daily average temperature (red dotted line) and cumulative rainfall (blue bar) during the 2020 and 2021 sorghum growing seasons of the four experimental sites of JG (Gongzhuling in Jilin province), LS (Shenyang in Liaoning province), HZ (Zhengzhou in Henan province), and GX (Xingyi in Guizhou province).

The daily average temperature in JG, LS, HZ, and GX sites from sowing to physiological maturity was 20.6°C, 21.9°C, 25.0°C, and 22.2°C in 2020, and 18.1°C, 19.8°C, 25.9°C, and 23.1°C in 2021. The cumulative rainfall during the entire sorghum growing season in 2020 and 2021 was 595.2 mm and 1112.1 mm at the JG site, 588.6 mm and 932.2 mm at the LS site, 477.8 mm and 1323.7 mm at the HZ site, and 1141.1 mm and 1259.8 mm at the GX site. Generally, the daily average temperature and cumulative rainfall increased with the increase in site latitude across the four experimental sites during both years (**Figure 2**).

The experiments included three treatments at each site, in which six widely and commonly planted sorghum varieties [Hongmaonuo 2 (A1), Jiniang 4 (A2), Liaonian 3 (A3), Jinnuo 3 (A4), Jiza 127 (A5), and Jinza 22 (A6)] were tested. Each sorghum variety was planted with two row spacing modes: 60 cm equidistant row spacing mode (R1) and wide (80 cm)-narrow (40 cm) row spacing mode (R2). Fox each row spacing mode, three in-row plant spacings were used (10 cm, 15 cm, and 20 cm), which roughly corresponds to plant densities of 83,000, 111,000, and 166,000 plants ha^-1^, respectively.

At each site, field plots were planted in a split-plot design with three replicates. The main plot was variety, the row spacing mode and in-row plant spacing were subplot and sub-sub plot, respectively. Each plot consisted of six rows of sorghum with an area of 18 m^2^ (5 m in length and 3.6 m in width). Cow manure was applied to each plot as the base fertilizer, and diammonium phosphate and potassium sulfate were broadcast for each plot before planting as the seed fertilizer, providing a source of 27 kg N ha^−1^, 69 kg P ha^−1^, and 75 kg K ha^−1^, respectively. In addition, at the five-leaf stage, urea was topdressed to provide 138 kg N ha^-1^. Sorghum was sown by a precision dibbler to plant at least five seeds per hole and thinned to retain two or three seedlings in one hole at the three-leaf stage. The seedlings were fixed to the specified density at the five-leaf stage. Irrigation was applied once before planting. Herbicides (premix of atrazine and metolachlor, Jintian Tech Co., Ltd., Liaoning, China) were sprayed after sowing but before emergence. Weeds in plots were removed by hand at the stem elongation stage. Pests including corn borer, aphid, cotton bollworm, slime insect, and other pests were well controlled by spraying insecticide (Beta-cyfluthrin, Shanghai Hulian Biological Pharmaceutical Co., Ltd., Xiayi, China) at the stem elongation and anthesis stages. Sorghum heads were covered with net bags to prevent bird damage during the grain-filling period. No obvious weed, pest or disease stress was observed at the two experimental seasons.

### 2.2 Sampling and measurement

Meteorological data, including the daily average temperature and cumulative rainfall, were obtained from the local meteorological station. Sorghum growth stages were recorded when over 50% of the sorghum plants in the field plot were within a specific period according to the standard method (Vanderlip and Reeves, 1972). The main growth stages of the six tested varieties across the four experimental zones in 2020 and 2021 are shown in **Supplementary Table S2**.

At harvest, about 12 m^2^ in the inner four rows of each plot were harvested to measure sorghum grain yield. A PM-8188A grain moisture analyzer (Tokyo, Japan) was used to determine the grain moisture content, which measured each kernel sample ten times and recorded the average moisture value. The grain of each plot was weighed and corrected to a water content of 15.5% for the final sorghum grain yield.

### 2.3 Data analysis

Analysis of variance (ANOVA) was done in R with the “agricolae” package to test the effect of the ecological zone, variety, plant density, row spacing, and their interaction on grain sorghum yield after the variance normality were tested using Shaprio-Wilk method. The mean sorghum yield values at each site for both years were tested using the least significant differences (LSD) test at the 5% (*P <* 0.05) probability level. Linear regression was conducted to assess the relationship between experimental site latitude and sorghum grain yield, plant density, and grain yield using a Loess regression curve. The linear regression and figures were carried out in R 4.0.5 (R Core Team, 2021).

## 3 Results

### 3.1 ANOVA analysis

A combined ANOVA analysis was conducted for the four ecological types. The effects of the year (Y), ecological type (E), variety (V), plant spacing [including row spacing mode (R) and plant density (D)], and their interactions on sorghum grain yield, growth duration, and plant height were analyzed for significance at the *P<0.05*, *P < 0.01*, or *P< 0.001* levels (**Table 1**). Results showed that E, V, and D significantly influenced sorghum grain yield (P<0.001). Meanwhile, significant effects of the E × V, E × D, and E × R interactions were also observed for sorghum grain yield (*P < 0.001*). However, no significant effects of the V × D interactions were detected. The Y, E, and V had a significant effect on both the growth duration and plant height. Notably, the R and Y× E interaction and the D and Y × D interaction significantly affected growth duration and plant height, respectively. Moreover, the variable Y did not have a significant effect on the sorghum grain yield. Therefore, this study pooled the Y variable with the E, V, D, and R effects on sorghum yield.

**Table 1:** Summary of the analysis of variance (ANOVA) of grain sorghum for the year (Y), ecological type (E), variety (V), row spacing mode (R), plant density (D), and their possible interaction on sorghum yield, growth duration, plant height, and the relative contribution of factors to sorghum yield variability during the two experimental years.

Source of variance	df	Yield			Growth duration				Plant height		
		F value	p-value	Contribution (%)	F value	p-value		Contribution (%)	F value	p-value	Contribution (%)
Year (Y)	1	0.77	0.38	0.02	604.13	0.00		0.98	2.71	0.00	0.24
Ecological type (E)	3	702.85	0.00	49.74	17965.00	0.00		87.23	41.77	0.00	1.57
Variety (V)	5	70.09	0.00	8.27	246.68	0.00		2.00	90.70	0.00	81.59
Row spacing mode (R)	1	0.50	0.48	0.01	6.23	0.01		0.01	2829.40	0.38	0.00
Plant density (D)	2	38.65	0.00	1.82	0.80	0.45		0.00	0.77	0.00	0.32
Y×E	3	37.48	0.00	2.65	999.08	0.00		4.85	27.66	0.00	2.12
Y×V	5	6.61	0.00	0.78	31.02	0.00		0.25	122.46	0.00	0.17
Y×R	1	0.14	0.71	0.00	7.33	0.01		0.01	5.76	0.19	0.01
Y×D	2	2.71	0.07	0.13	0.48	0.62		0.00	1.75	0.03	0.04
E×V	15	17.94	0.00	6.35	122.20	0.00		2.97	3.40	0.00	8.58
E×R	3	3.58	0.01	0.25	1.36	0.25		0.01	99.15	0.79	0.01
E×D	6	5.46	0.00	0.77	0.32	0.93		0.00	0.35	0.13	0.06
V×R	5	0.73	0.60	0.09	1.06	0.38		0.01	1.66	0.20	0.04
V×D	10	1.52	0.13	0.36	0.93	0.50		0.02	1.47	0.51	0.05
R×D	2	0.98	0.37	0.05	0.35	0.70		0.00	0.92	0.29	0.01
Y×E×V	15	4.88	0.00	1.73	22.30	0.00		0.54	1.23	0.00	0.92
Y×E×R	3	2.12	0.10	0.15	1.67	0.17		0.01	10.60	0.52	0.01
Y×E×D	6	5.59	0.00	0.79	0.53	0.78		0.01	0.76	0.92	0.01
Y×V×R×	5	1.24	0.29	0.15	0.55	0.74		0.00	0.33	0.90	0.01
Y×V×D	10	0.58	0.83	0.14	0.51	0.89		0.01	0.32	0.93	0.02
Y×R×D	2	0.04	0.96	0.00	0.15	0.86		0.00	0.43	0.55	0.01
E×V×R	15	0.62	0.86	0.22	0.75	0.73		0.02	0.60	0.13	0.12
E×V×D	30	0.95	0.54	0.68	0.77	0.80		0.04	1.43	0.94	0.11
E×R×D	6	0.35	0.91	0.05	0.46	0.84		0.00	0.62	0.68	0.02
V×R×P	10	1.61	0.10	0.38	0.47	0.91		0.01	0.66	0.49	0.05
Y×E×V×R	15	0.97	0.49	0.34	0.70	0.78		0.02	0.94	0.92	0.05
Y×E×V×D	30	1.07	0.36	0.76	0.49	0.99		0.02	0.53	0.72	0.14
Y×E×R×D	6	0.43	0.86	0.06	0.31	0.93		0.00	0.84	0.40	0.04
Y×V×R×D	10	0.91	0.52	0.22	0.35	0.97		0.01	1.04	0.99	0.02
E×V×R×D	30	1.16	0.26	0.82	0.66	0.92		0.03	0.27	0.97	0.10
Y×E×V×R×D	30	0.31	0.40	1.00	0.29	1.00		0.01	0.5	0.1	0.22
Error	574										
Total	863										

Y, Year; E, Ecotype; V, Variety; R, Row spacing mode; D, Plant density.

We could also see from **Table 1** that there was great difference in the relative contribution of each variable to the variances of sorghum grain yield, growth duration, and plant height. In case of grain yield, ecological type (E) contributed the most to its variance (49.7%), followed by the variety (V, 8.27%) and plant density (D,1.82%), and row space (R) contributed only 0.01%. Among interactions, the largest contributor was E × V (6.37%), then was year (Y) × E (2.65%), Y×E×V (1.73) and Y×E×V×R×D (1%). All other interactions contributed respectively less than 1% of the total grain yield variance.

In case of growth duration, among main effect factors, the largest contribution of its variance was still ecological type (87.23%), followed by variety (2.0%) and year (0.98%), while almost no contribution came from row space and plant density. Among interactions, Y×V contributed the most to the total variance of growth duration (4.85%), followed by E×V (2.97%). All other interaction contributed respectively less than 1% to the total variance.

As for plant height, it was mainly affected by genotype, contributed 81.59% of the total variance, followed by ecological type (1.57%). Year and plant density has minor effect to plant height, contributed only 0.24% and 0.32% respectively, and no effect on plant height was observed from row space. Among interactions, E × V was the largest contributor to the total variance of plant height (8.58%), then was Y×E (2.12%),

From the above analysis, it could be concluded that sorghum grain yield was mainly influenced by environment, genotype and their interaction; the interaction of genotype, environment and agronomic practices contributed the highest yield. Basically, the growth duration was influenced by ecological type, while plant height was main influenced by genotype.

As shown in **Table 2**, ANOVA for each ecological type revealed that the yield performance over the two experimental years at each site was different (**Table 2**). The yield difference of two years at JG and GX was not significant (*P>0.05*); nevertheless, at LS and HZ, it was significant (*P<0.01*). The yield differenceof six varieties were found to be highly significant (*P<0.01*) at the JG and GX site, but not at the LS and HZ site. At each of the four sites, the responses to the row spacing mode and the in-row plant spacing (plant density) varied. For the row spacing mode, the yield effect was not significant (*P>0.05*) at JG and HZ but very significant (*P<0.01*) at LS and GX. For the in-row plant spacing (plant density), the yield effect was extremely significant (*P<0.01*) at three sites (except the LS site). The performance of various interactions was also different at different sites. For example, effect of variety × row spacing mode on yield was only significant at the GX site, while variety ×plant density interaction was significant only at the HZ site. Plant density × row spacing mode was not significant at all four sites, interactions of variety × row spacing ×plant density was significant (*P<0.05*) only at the LS site.

**Table 2:** Significant *P* value of ANOVA in different sites and years.

Source of Variance	Degree of Freedom	LS	JG	HZ	GX
Year (Y)	1	0.0000	0.1178	0.0000	0.4276
Variety (V)	5	0.5325	0.0057	0.0788	0.0000
Ea	5				
Row spacing mode (R)	1	0.0468	0.8271	0.2309	0.0003
V×R	5	0.3658	0.8844	0.9876	0.0001
Eb	6				
Plant density (D)	2	0.2905	0.0002	0.0000	0.0000
V×D	10	0.0837	0.8438	0.0176	0.0788
R×D	2	0.5412	0.5875	0.9451	0.2391
V×R×D	10	0.0255	0.4824	0.1259	0.3513
Ec	24				

Y, Year; V, Variety; R, Row spacing mode; D, Plant density; LS, Shenyang in Liaoning province; JG, Gongzhuling in Jilin province; HZ, Zhengzhou in Henan province; GX, Xingyi in Guizhou province. Ea, Eb, and Ec indicate error for the main plot, subplot, and sub-sub plot, respectively.

### 3.2 Effect of ecological zone on sorghum yield and growth duration

**Figure 3:**
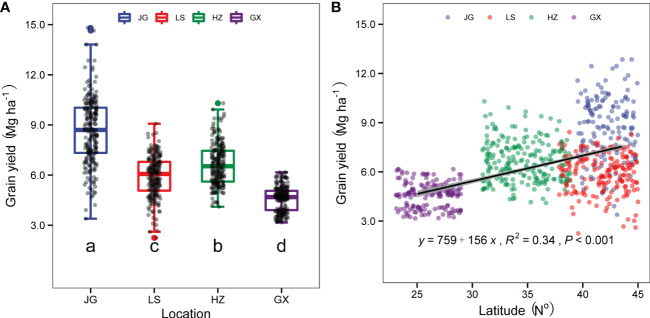
The average sorghum grain yield of the four experimental sites, JG site (blue box), LS site (red box), HZ site (green box), and GX site (purple box) **(A)**; and the relationship between sites latitude and sorghum grain yield **(B)**. Different letters under vertical bars indicate significant differences (p<0.05) within the sites. Linear regression analysis is used to determine the significant line. The shaded area indicates the 95% confidence interval of the regression line.

Sorghum grain yield was significantly affected by the ecological zones (**Figure 3A**). Across the six sorghum varieties and for the two experimental years, the Gongzhuling (JG) site recorded the highest average yield of 8740.9 kg ha^-1^, followed by Zhengzhou (HZ), Shenyang (LS), and Xingyi (GX), with average yields of 6627.0 kg ha^-1^, 5872.0 kg ha^-1^, and 4584.8 kg ha^-1^, respectively. The sorghum grain yield of six varieties across three plant densities for both years had an overall uptrend with increasing site latitude (**Figure 3B**).

**Figure 4:**
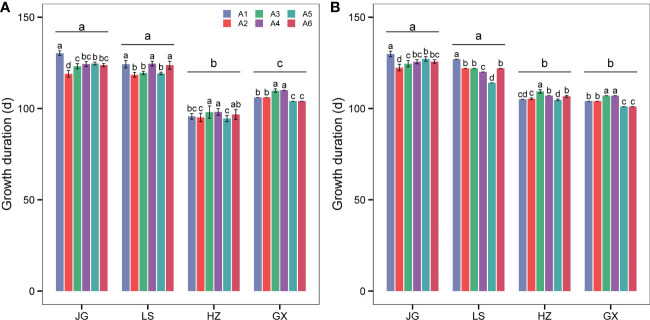
The average growth duration of six test varieties: Hongmaonuo 2 (A1-blue box), Jiniang 4 (A2-red box), Liaonian 3 (A3-green box), Jinnuo 3 (A4-purple box), Jiza 127 (A5-dark green box), and Jinza 22 (A6-dark red box) at JG, LS, HZ, and GX site in 2020 **(A)** and 2021 **(B)**. Different letters up error bars and the top horizontal line indicate significant differences (p<0.05) within the varieties and ecotypes, respectively.

As shown in **Figure 4**, the growth duration varied significantly among the four ecological zones in 2020 (**Figure 4A**) and 2021 (**Figure 4B**), ranging from 116-132 d, 114-127 d, 93-110 d, and 101-110 d, at JG, LS, HZ, and GX, respectively. The average growth duration of JG was the highest (125.0 d), followed by LS (121.4 d), GX (105.3 d), and HZ (101.3 d). The average growth duration of the JG and LS sites was significantly higher than that of the HZ and GX sites. There was no significant growth duration difference between the JG and LS sites or the HZ and GX sites.

### 3.3 Effects of variety on yield and plant height

**Figure 5:**
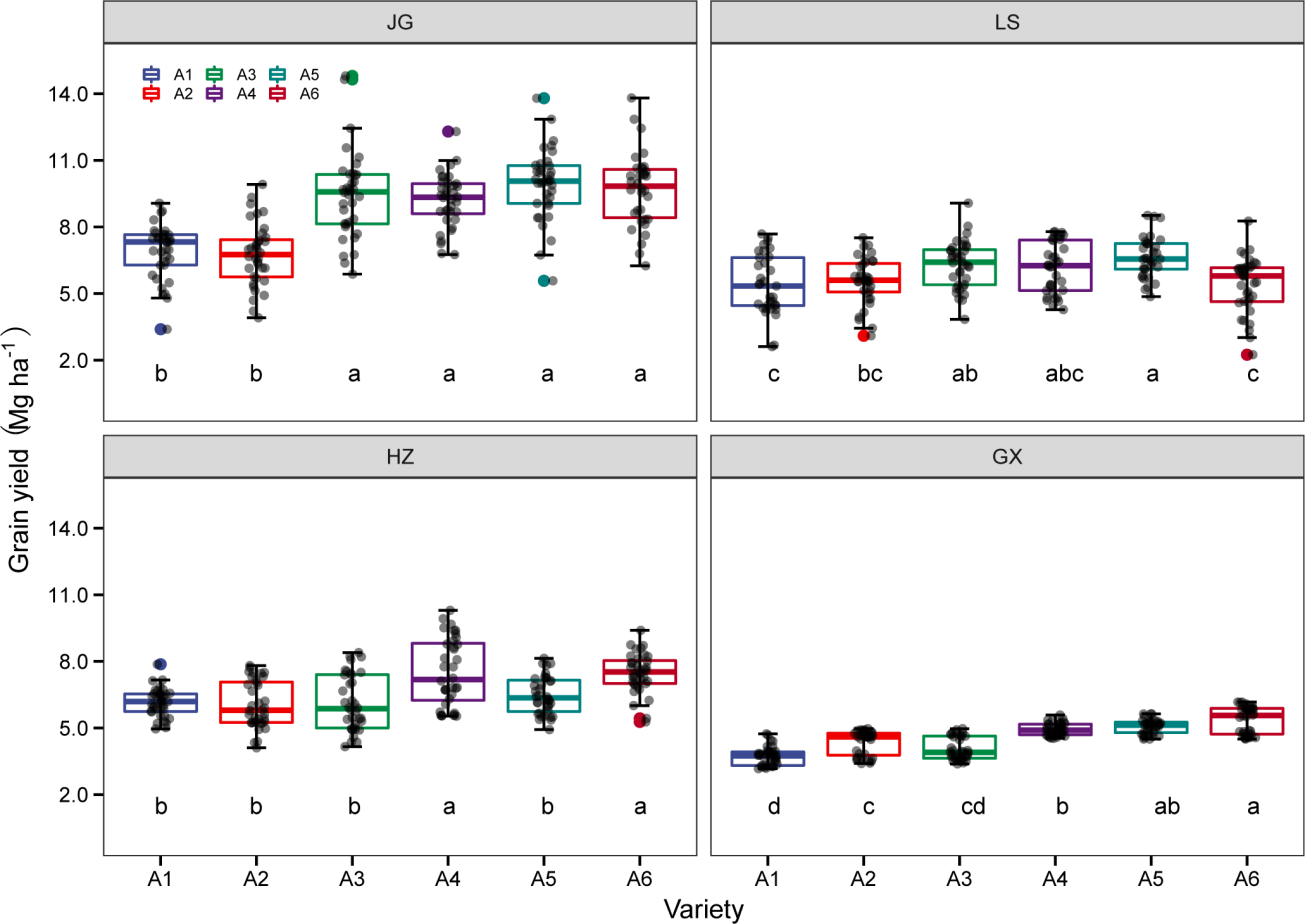
The average sorghum grain yield of six tested varieties: Hongmaonuo 2 (A1-blue box), Jiniang 4 (A2-red box), Liaonian 3 (A3-green box), Jinnuo 3 (A4-purple box), Jiza 127 (A5-dark green box), and Jinza 22 (A6-dark red box) at JG, LS, HZ, and GX site. Different letters up error bars and the top horizontal line indicate significant differences (p<0.05) within the varieties.

As shown in **Figure 5**, the grain yield of six sorghum varieties differed significantly at four experimental sites. For the two experimental years, the average yield of A6 (Jinza 22) and A5 (Jiza 127) was 10182.3 kg ha^-1^ and 9933.8 kg ha^-1^ at the JG site and 5364.8 kg ha^-1^and 5061.6 kg ha^-1^ at the GX site, respectively. This was significantly higher than the average value of A3 (Liaonian 3), A4 (Jinnuo 3), A1 (Hongmaonuo 2), and A2 (Jiniang 4) (**Figure 5**). At the LS site, the average yields of A5 (Jiza 127) and A3 (Liaonian 3) were 6457.3 kg ha^-1^, and 6306.8 kg ha^-1^, respectively, which were significantly higher than those of A1 (Hongmaonuo 2), A2 (Jiniang 4), A4 (Jinnuo 3), and A6 (Jinza 22). At the HZ site, A4 (Jinnuo 3), and A6 (Jinza 22) had the highest yields of 7514.2 kg ha^-1^ and 7477.8 kg ha^-1^ across two experimental years, followed by A5 (Jiza 127), A1 (Hongmaonuo 2), A3 (Liaonian 3), and A2 (Jiniang 4). Among all ecological zones, A6 had the highest average yield of 7109.0 kg ha^-1,^ followed by A5 and A4 with 6984.6 kg ha^-1^ and 6931.6 kg ha^-1^, respectively; these results were not significantly different from A6 (Jinza 22). A3 (Liaonian 3) had a yield of 6478.6 kg ha^-1^, which was significantly lower than A6 (Jinza 22), A5 (Jiza 127), and A4 (Jinnuo 3). A2 (Jiniang 4) and A1 (Hongmaonuo 2) had a yield of 5680.9 kg ha^-1^ and 5530.8 kg ha^-1^, respectively, which was significantly lower than the other four varieties.

**Figure 6:**
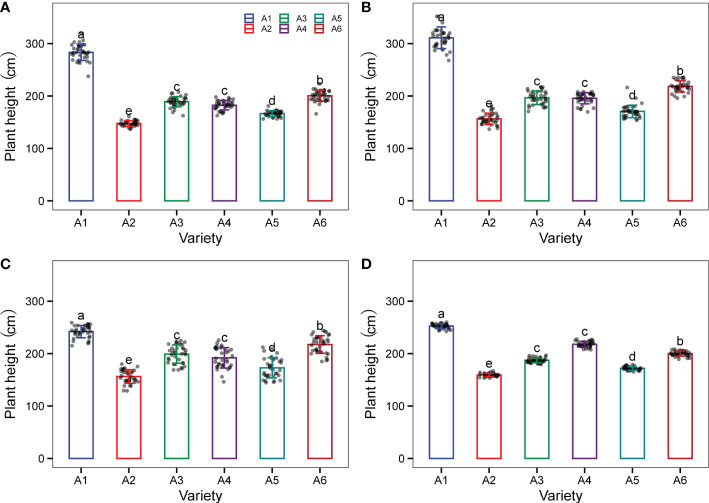
The plant height of the six test varieties: Hongmaonuo 2 (A1-blue box), Jiniang 4 (A2-red box), Liaonian 3 (A3-green box), Jinnuo 3 (A4-purple box), Jiza 127 (A5-dark green box), and Jinza 22 (A6-dark red box) at JG site **(A)**, LS site **(B)**, HZ site **(C)**, and GX site **(D)** across the two experimental years. Different letters up error bars and the top horizontal line indicate significant differences (p<0.05) within the varieties.

As shown in **Figure 6**, the plant heights of six sorghum varieties differed significantly across four ecological zones during the two experimental years.The plant height of A1 (Hongmaonuo 2) in all cases was the highest (272.2 cm), followed by A6 (Jinza 22), A4 (Jinnuo 3), and A3 (Liaonian 3), which were 209.0 cm, 197.0 cm, and 193.0 cm, respectively, and significantly lower than A1. The plant height of A5 (Jiza 127) was 170.4 cm, significantly lower than the aforementioned four varieties but significantly higher than the plant height of A2 (Jiniang 4, 154.9 cm).

### 3.4 Effects of plant population on grain yield

**Figure 7:**
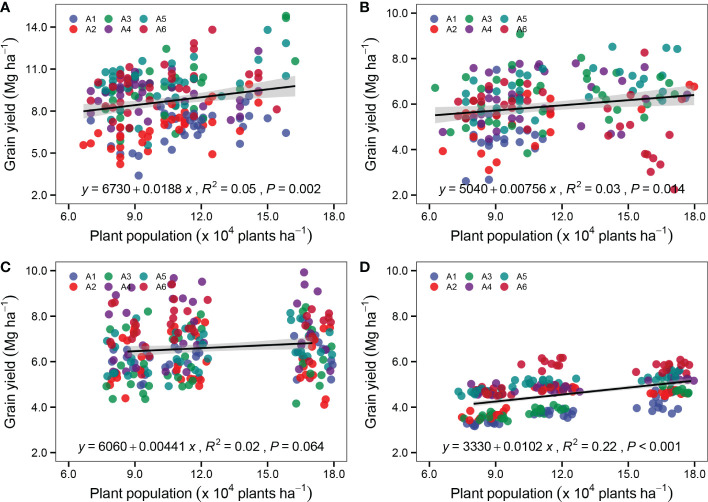
Relationship between plant population and sorghum grain yield of six varieties Hongmaonuo 2 (A1-blue circle), Jiniang 4 (A2-red circle), Liaonian 3 (A3-green circle), Jinnuo 3 (A4-purple circle), Jiza 127 (A5- dark green circle), and Jinza 22 (A6-dark red circle) at JG site **(A)**, LS site **(B)**, HZ site **(C)**, and GX site **(D)**. Linear regression analysis is used to determine the significant line. The shaded area indicates the 95% confidence interval of the regression line.

Significant linear relations were observed between the plant population and the sorghum grain yield of six sorghum varieties in both years; this indicated that the four experimental sites tested in the current study could support a sorghum plant population greater than 83,000 plants ha^-1^ (**Figure 7**). Increasing the plant population from 83,000 to 166,000 plants ha^-1^ increased the sorghum yield in the experimental sites of JG by 188.0 kg ha^-1^ per 10,000 plants (**Figure 7A**), LS by 75.6 kg ha^-1^ per 10,000 plants (**Figure 7B**), and GX by 102.0 kg ha^-1^ per 10,000 plants (**Figure 7D**). However, increasing the plant population led to no significant yield increase at the HZ site (**Figure 7C**).

**Figure 8:**
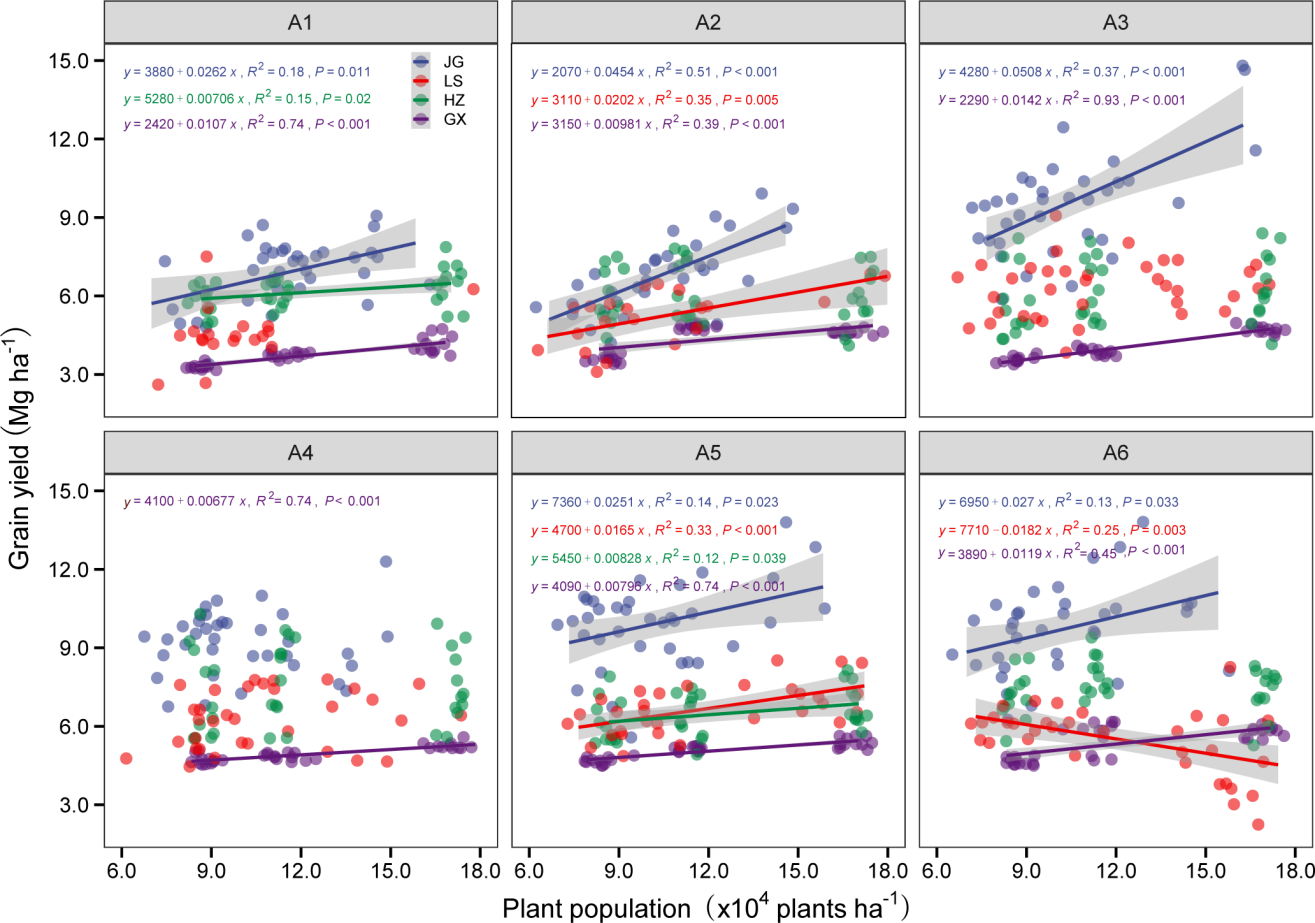
Relationship between plant population and sorghum grain yield at JG site (blue circle), LS site (red circle), HZ site (green circle), and GX site (purple circle) of six tested varieties Hongmaonuo 2 (A1), Jiniang 4 (A2), Liaonian 3 (A3), Jinnuo 3 (A4), Jiza 127 (A5), and Jinza 22 (A6). Linear regression analysis is used to determine the significant line, only showing lines with a significant difference. The shaded area indicates the 95% confidence interval of the regression line.

To further identify the yield of six sorghum varieties in response to the plant population at four experimental sites, the relationships between the plant population at harvest and the yield of each sorghum variety were analyzed at the four experimental sites (**Figure 8**). The variety A1 (Hongmaonuo 2) showed significant linear relationships between the plant population and the sorghum grain yield at three experimental sites (except LS site), whereas the variety A5 (Jiza 127) had significant linear associations at four experimental sites. The slope at the JG site was the highest, followed by the LS, GX, and HZ sites. A2 (Jiniang 4) and A6 (Jinza 22) had similar trends at the JG, LS, and GX sites. In contrast, with the increasing plant population, A3 (Liaonian 3) had a steep grain yield increase at the JG and GX sites. It is worth noting that A3 had the maximum slope at the JG site among six varieties; A4 (Jinnuo 3) only had a smaller increase at the GX site with a slope of 67.7 kg ha^-1^ per 10,000 plants.

### 3.5 Effects of plant spacing on grain yield

#### 3.5.1 Row spacing mode

**Figure 9:**
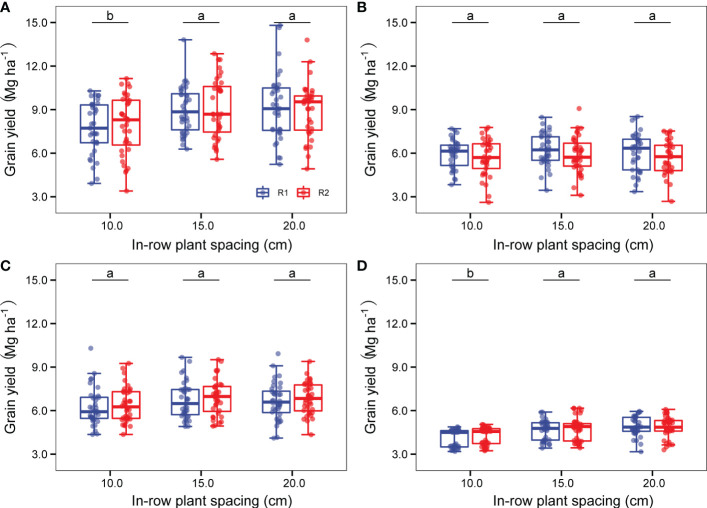
The average sorghum grain yield of 60 cm equidistant row spacing mode (R1) and wide (80 cm)-narrow (40 cm) row spacing mode (R2) at 83,000 plants ha^-1^(D1), 111,000 plants ha^-1^ (D2), and 166,000 plants ha^-1^ (D3) of JG site **(A)**, LS site **(B)**, HZ site **(C)**, and GX site **(D)** during the two experimental years. Different letters up error bars and the top horizontal line indicate significant differences (p<0.05) within the plant densities.

As shown in **Figure 9** and **Supplementary Table S3**, although there were no significant differences in yield between the two row spacing modes, there were markedly different tendencies among experimental sites and in-row plant spacings. At JG and GX sites (**Figures 9A–D**), sorghum planted in a wide-narrow row planting mode (R2) increased sorghum yield by 2.4% and 3.8% at 83,000 plants ha^-1^ (D1), 1.1% and 2.3% at 111,000 plants ha^-1^ (D2), but decreased sorghum yield by 2.2% and 2.0% at 166,000 plants ha^-1^ (D3), compared to equidistant row planting mode (R1). At the LS site (**Figure 9B**), the wide-narrow row spacing mode decreased sorghum yield by 6.3% at D1, 5.7% at D2, and 8.4% at D3, respectively, compared to the equidistant row spacing mode (R1). At the HZ site (**Figure 9C**), a wide-narrow row spacing mode (R2) increased sorghum yield by 2.8%-3.1% across three plant densities compared to the equidistant row spacing mode.

#### 3.5.2 In-row plant spacing

Sorghum yield increased with increasing planting density, with 0.7 Mg ha^-1^ increasing with equidistant row spacing mode (R1) and 0.59 Mg ha^-1^ increasing with wide-narrow row spacing mode (R2) across four experimental sites of six sorghum varieties in both years. At the JG and GX sites, the R1 had a greater advantage in sorghum yield benefit than that of the R2 (+1.51 Mg ha^-1^ vs +1.14 Mg ha^-1^ at JG, +0.68 Mg ha^-1^ vs +0.58 Mg ha^-1^ at GX) while planting density increased from 83,000 plants ha^-1^ (D1) to 111,000 plants ha^-1^ (D2) and 166,000 plants ha^-1^ (D3) (**Figure 9**).

#### 3.5.3 Effect of plant spacing configuration

It was shown in **Supplementary Figure S1** that in all cases (48) of configuration (row spacing mode and in-row plant spacing) in four sites during the two experimental years, only one case had a higher yield under wide-narrow row mode (R2) in one site (HZ) in one year. Otherwise, higher yields were observed under the equidistant row spacing mode (R1). This indicates that equidistant row spacing mode (R1) is more advantageous than wide-narrow row spacing mode (R2) for mining grain yield potential.

### 3.6 Interaction of variety-ecological types-plant spacing

#### 3.6.1 Interactions of year with ecological type, variety, and in-row plant spacing

**Figure 10:**
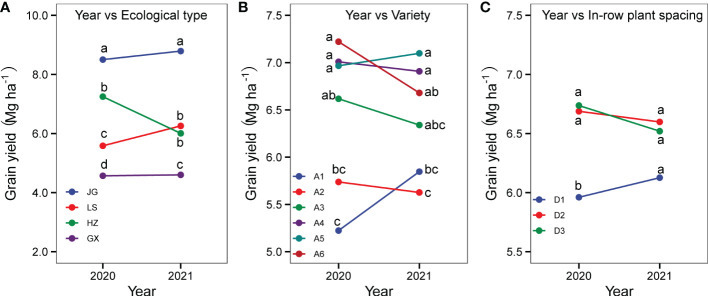
Interaction between year and ecological types **(A)**, variety **(B)**, and in-row plant spacing **(C)**. Different letters under vertical bars indicate significant differences (p<0.05) within the year.

As shown in **Figure 10**, the year strongly interacted with ecological type, variety, and plant spacing (*P<0.01*); this indicates that the impact of these factors varied by year. The interaction of year and ecological type revealed that the grain yield change model between two years differed (**Figure 10A**); at LS, grain yield increased significantly in 2021 compared to 2020. Grain yield at HZ has decreased in 2021. However, at GX and JG, the yield difference was not significant. The year-variety interaction demonstrated various ways in which the mean yield of each variety responded to the year (**Figure 10B**). The mean yields of A1 (Hongmaonuo 2) and A5 (Jiza 127) increased by 11.91% and 1.28%, respectively. The mean yields of A2 (Jiniang 4), A3 (Liaonian 3), A4 (Jinnuo 3), and A6 (Jinza 22) decreased by 1.72%, 3.66%, 1.68%, and 8.12%, respectively. The sorghum yield under low density (in-row plant spacing of 15-20 cm) was higher in 2021 than it was in 2020, according to the year-in-row plant spacing interaction (**Figure 10C**); the inverse was true for grain yield under a higher density (in-row plant spacing of 10 cm).

#### 3.6.2 Interactions of ecological types with variety, row spacing mode and in-row plant spacing

**Figure 11:**
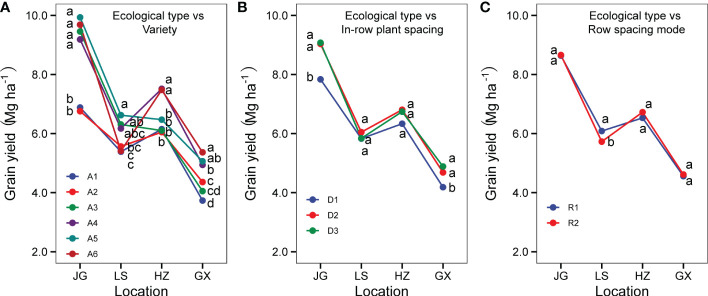
Interaction between ecological type between variety **(A)**, in-row plant spacing **(B)**, and row spacing mode **(C)**. Different letters under vertical bars indicate significant differences (p<0.05) within the site.

As shown in **Figure 11**, the genotype effect was influenced by ecological types, and the relative performance of six varieties was different in four ecological sites. At JG, the yield performance of six varieties varied widely and was divided into two groups: the varieties A1 (Hongmaonuo 2) and A2 (Jiniang 4) that were developed respectively in the southwest zone and spring/summer sowing zone had a lower yield than the other four varieties (developed in the spring sowing zones). At LS, the mean yield differences among six varieties were not larger compared to the JG site. The variety A5 (Jiza127) had the highest grain yield, with the same relative performance. The relative yield performance of A6 (Jinz22) was different from that at JG, ranked 2 among six varieties at JG, but ranked fifth at LS. The other four varieties had a similar mean yield rank. Six varieties were divided into two groups: the (1) high yield group, including A3 (Liaonian 3), A4 (Jinnuo3), and A5 (Jiza127); and(2) low yield group, including A1 (Hongmaonuo 2), A2 (Jiniang 4), and A6 (Jinz22). At HZ, yield level was significantly higher than that at LS; six varieties were divided into three groups: the (1) high yield group, including A4 (Jinnuo3), A6 (Jinz22); (2) middle yield, including A5 (Jiza127); and (3) low yield group, including A1 (Hongmaonuo 2), A2 (Jiniang 4), and A3 (Liaonian 3). The total mean grain yield was the lowest at GX. The six varieties were also divided into two groups as per their grain yield: group 1 included A4 (Jinnuo3), A5 (Jiza127), and A6 (Jinz22), with higher yields; group 2 included A1 (Hongmaonuo 2), A2 (Jiniang 4), and A3 (Liaonian 3), with lower yields (**Figure 11A**).

As shown in **Figure 11B**, the ecological types and in-row plant spacing interaction showed that the responses of four sites to in-row plant spacing were different. At JG and GX, the grain yield under 10 cm (D3) and 15 cm (D2) in-row plant spacing was significantly higher than that under 20 cm (D1); the grain yield under 10 cm (D3) was slightly higher than that under 15 cm (D2) in-row plant spacing, but the difference was not significant. While at LS, grain yield under 15 cm (D2) in-row plant spacing was higher than that under 10 cm (D3), and 20 cm (D1), although the difference was not significant. At HZ, grain yield under plant spacing of 10 cm (D3) was the highest, with no significant difference under plant spacing of 15 cm (D2) and 20 cm (D1).

As shown in **Figure 11C**, the ecological types and row spacing mode interaction showed that the response in four sites to row mode was not the same. At JG, HZ, and GX, the grain yield under uneven row spacing was a little higher than under equidistant row spacing but didn’t reach a significant level. While at LS, equidistant row spacing mode had a significantly higher yield than wide-narrow row spacing mode. This showed, in spring sowing late-maturing zone with sporadically limited rainfall, uneven-row-space was not suitable for rainfed sorghum production.

#### 3.6.3 Three-factor interactions of year-ecological types-variety and year-ecological types-in-row planting spacing

**Figure 12:**
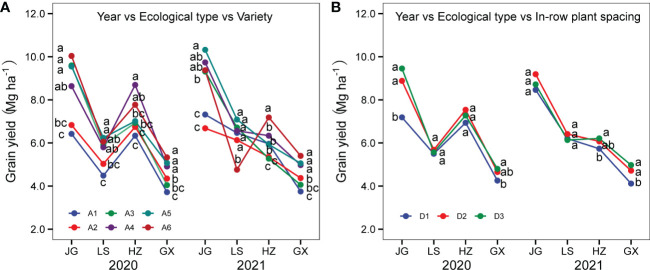
Interactions of year-ecological type-variety **(A)** and year-ecological type-in-row plant spacing **(B)**. Different letters under vertical bars indicate significant differences (p<0.05) within the site and experimental year.

As shown in **Figure 12A**, the yield performance of six varieties differed by year and ecological type. For example, variety A1 (Hongmaonuo 2) had the lowest grain yield in all four sites in 2020 and, in 2021, ranked third at GX and ranked fourth at LS. The variety A6 (Jinza22) ranked first at JG and GX in 2020 and first at HZ and GX in 2021, with the yield ranking first in 2020 but lowest at LS in 2021. The variety A6 (Jinza22) ranked first at JG and GX in 2020 and at HZ and GX in 2021, of which yield ranked in group one in 2020 but the lowest in 2021 at LS. Based on the factors of variety, year, and site, the best combination was A5 (Jiza127)-JG-2021, followed by A4 (Jinnuo3)- JG-2021 and A6 (Jinza22)-JG-2020.

Results in **Figure 12B** reveal that the yield performance of three in-row plant spacings varied by year and ecological type. At JG, the degree of response to plant spacing was higher in 2020 than in 2021. Yield under in-row plant spacing of 10 cm (D3) was the highest in 2020, but in 2021, intra-row plant spacing of 15 cm (D2) had the highest yield. At LS, the difference in mean grain yields under three plant spacings was not significant. It had the following yield ranking: 15 cm (D2)>10 cm (D3)>20 cm (D1) in 2020; 10 cm (D3)>15 cm (D2)>20 cm (D1) in 2021. At HZ, grain yields under plant spacings of 15 cm (D2) and 10 cm (D3) were significantly higher than those under the plant spacing of 20 cm (D1) in both years. However, the highest yield was observed under 15 cm (D2) in 2020, while 10 cm (D3) had the highest yield in 2021. The difference between 15 cm (D2) and 10 cm (D3) was not significant in either year. At GX, the response to plant spacing was similar in both years.

## 4 Discussion

Sorghum grain yield among the four experimental sites varied greatly, with yield at the Gongzhuling (JG) site being the highest, followed by Zhengzhou (HZ) and Shenyang (LS), and yield at Xingyi (GX) being the lowest. The JG site is located in the spring-sown early-maturing zone, which is a traditional zone for grain sorghum production in China (Diao, 2017). Since the majority of grain sorghum is planted in arid and semi-arid regions, and sorghum is generally grown under rain-fed conditions, yield in the spring-sown early-maturing zone averaged 7.2 Mg ha^-1^, followed by the summer-sown zones (6.3 Mg ha^-1^) (Li et al., 2018). However, it should be noted that although the summer-sown zone had abundant precipitation and heat units during the sorghum growing season, it is typically a double cropping system, and the varieties are mainly summer-sown early-maturing varieties, which are similar to the varieties in the spring-sown early-maturing zone (Pei et al., 2017). Sorghum yield in the spring-sown late-maturing zone averaged 5.0 Mg ha^-1^ according to the Liaoning Statistic Yearbook (Chen et al., 2016). The GX site in Guizhou province, China, is a typical southern zone with abundant rainfall and heat units; however, the condition of light resources is weaker than that of the other three zones (Zhang et al., 2009; Li et al., 2013). This region is a major center for liquor brewing in the country, and sorghum is divided into contiguous and scattered planting modes (Jiang et al., 2019; Su et al., 2020). Generally, in the contiguous planting mode, varieties are carefully planted according to the requirements of liquor-related enterprises. In contrast, local farmers use the technique of scattered planting of sorghum on the hills or terraces with poor soil conditions and the ability to retain fertilizer and water (Gao et al., 2010). Therefore, sorghum yield in this zone averaged 4.2 Mg ha^-1^ based on the China Statistical Yearbook of 2019, which is significantly lower than that of the other three zones. In this study, the average yield of the four experimental sites was higher than the local yield by 5.2%-21.4%, indicating that sorghum yield could be significantly improved through reasonable cultivar selection and management practices.

Variety is one of the most important factors affecting sorghum yield. Zone-specific field management practices are often based on varieties, and selecting a sorghum variety is the top priority in formulating high-yield strategies. Maturity, fitness (or resistance), and productivity are the most important considerations for variety selection. In general, the yield of late-maturing varieties is higher than that of early-maturing varieties. However, early-maturing varieties are selected to ensure normal maturity of sorghum due to the limitation of accumulated heat units, especially in the spring-sown early-maturing zone (Gao et al., 2022). As mentioned in the introduction, most sorghum is planted in arid and semi-arid regions. The drought resistance of varieties is an important consideration for obtaining a high and stable grain yield. However, the drought resistance of varieties is usually difficult to estimate quantitatively. The regional test control variety is often used as a reference. In the present study, the variety A6 (Jinza 22) had the highest average yield across four experimental sites, indicating its wide adaptability. For the four experimental sites, in the spring-sown early-maturing zone (JG) and the southern zone (GX), A6 (Jinza 22) and A5 (Jiza 127) have the best yields; this indicates that the early-maturing and wide-adaptive varieties in the aforementioned two regions should be given priority for variety selection. In the spring-sown late-maturing zone (LS), A5 (Jiza 127) and A3 (Liaonian 3) had the best yields; in the summer-sown zones, A4 (Jinnuo 3) and A6 had the highest yields. These findings suggest that late-maturing varieties may be chosen to obtain higher yields in ecological zones with adequate accumulated heat units.

Cultivation with high-density planting is currently one of the most important management practices to increase cereal crop yield, and the suitability of varieties for high density is becoming an important criterion in variety selection. In field production, the effect of densification on sorghum yield is jointly affected by growing conditions and varieties in the cultivation area. From the perspective of growing conditions, zones with better light, accumulated heat units, and soil conditions are the traditional high-yield zones, where densification often causes an increase in yield, and the plant density such an environment can support is higher than that of medium and low-yield zones. JG is the traditional high-yield zone; sorghum yield increased significantly while planting density increased from 83,000 plants ha^-1^ to 166,000 plants ha^-1^ across the six test varieties. Shen et al. (2016) documented that the optimum plant density in this region could reach 200,000 plants ha^-1^. Therefore, sorghum yield can be further increased by increasing plant density in the high-yield zone. According to (Xiao et al., 2018) and (Zhang et al., 2016), the optimal plant density in the medium-yield zones of LS and HZ is 135,000 plants ha^-1^ and 105,000 plants ha^-1^, respectively. These values are compatible with the findings of the current study and show that the medium-yield zone can be properly densified under the current conditions. In the low-yield zone, the sorghum yield increase caused by densification is the lowest, with the optimum plant density in these zones ranging between 50,000 plants ha^-1^ to 100,000 plants ha^-1^. However, since sorghum has a large capacity to produce tillers, the optimum planting density for grain yield is wide and depends largely on soil moisture content and variety maturity (Abunyewa et al., 2010; Adam et al., 2020). The yield of varieties with strong planting density tolerance typically has a better response to densification, but this response is affected by growing conditions as well (Baumhardt and Howell, 2006). In this study, A1 (Hongmaonuo 2) and A5 (Jiza 127) had a significant linear relationship with yield and plant density across four experimental sites. The slope of the linear line is higher in the high-yield zone than that in the medium and low-yield zone. A2 (Jiniang 4) and A6 (Jinza 22) had similar trends to A1 (Hongmaonuo 2) and A5 (Jiza 127). It is worth noting that A3 (Liaonian 3) only had a better yield and plant density response in the spring-sown early-maturing zone and the southern zone. Similarly, A4 (Jinnuo 3) showed a better yield and plant density response trend in the southern zone, which was consistent with previous studies (Wang et al., 2019; Zhang et al., 2019); they found that A4 (Jinnuo 3) had strong adaptability and high productivity in the early-maturing zone. Therefore, while deciding on densification, variety and maturity traits should be considered initilly. Variety and density tolerance should then be considered in combination with the growing conditions of the region to further boost sorghum productivity.

Plant-spacing configuration is a more recent planting practice developed with densification. In field cereal crops such as maize, narrowing the row spacing or planting with a wide-narrow row pattern could improve the interception of light and increase dry matter accumulation and grain yield (Bernhard and Below, 2020a; Bernhard and Below, 2020b). Maiga (2012) indicated that narrow row spacing could achieve a 3%-14% sorghum yield benefit compared with wide row spacing. However, it was found that the wide-narrow row pattern was not effective in increasing sorghum yield compared with equal-row planting (Sanabria et al., 1995; Steiner, 1986). The differences between the cited research results may be closely related to the selection of regional growing conditions, varieties, and planting density. In the current study, in the spring-sown early-maturing zone and the southern zone, compared with the equal-row pattern, the wide-narrow row pattern increased sorghum yield at a low planting density and caused a sorghum yield penalty at a high planting density. There are two possible reasons. Firstly, the JG site is the traditional high-yielding zone with high optimum planting density, but the wide-narrow row pattern at high planting density would result in low interplant spacing. Low interplant spacing results in stunted plant growth and yield reduction, which is a common phenomenon in maize (Sarlangue et al., 2007; Ruffo et al., 2015). Secondly, the southern zone is not suitable for high planting density due to the limitation of light resources (Zhang et al., 2010), and the wide-narrow row pattern will further reduce the interplant spacing compared with the equal-row pattern at high planting density; this reduction will affect individual plant growth and even cause premature senescence, which leads to a yield penalty. In the spring-sown late-maturing zone (LS) in the present study, the wide-narrow row pattern caused sorghum yield reduction under various planting densities; this may be due to the lack of cumulative rainfall during the early growth stage of sorghum. The wide-narrow row pattern also increased the sorghum canopy’s light transmittance (Fernandez et al., 2012; Timlin et al., 2014). The direct sunlight exposure could lead to soil water loss (Kugedera et al., 2022), resulting in early droughts and sorghum yield reduction. In the summer-sown zones (HZ) in the current study, the sorghum yield of the wide-narrow row pattern is higher than that of the equal-row pattern at various planting densities. This may be due to the sufficient rainfall and light resources in the early sorghum growth stage in this zone (Yang et al., 2022). The wide-narrow row pattern reduced sorghum canopy closure and increased the canopy’s ventilation and light transmission; these changes reduced the risk of pests and diseases during the late sorghum growth stage. Overall, in the high-yield zone with high optimum planting density and insufficient rainfall zones, priority should be given to sowing in the equal-row pattern. With insufficient light resources and low optimum plant density in the summer-sown zone with sufficient rainfall, the wide-narrow row pattern is better than the equal-row pattern.

Generally, the G×E×M interaction is a relatively complex but important issue. This study further confirms that the cultivation environment has the greatest impact on sorghum yield, followed by the varieties. Management practices have the smallest impact on sorghum yield. Therefore, the growing conditions should be the priority in sorghum production, and varieties with good adaptability and high productivity should be selected and formulated with feasible management practices (row spacing, in-row plant spacing, etc.) to achieve a stable and high yield of sorghum. However, a broader scope and further exploration trending toward a steady increase in sorghum grain yield are needed. For example, the mechanism of the yield gap between similar ecological zones such as JG and LS must be thoroughly examined. Understanding this will help us further boost sorghum yield in major sorghum growing zones in China.

## 5 Conclusion

Sorghum yield was significantly influenced by ecological types, varieties, and plant spacing configuration, with ecological types accounting for the largest share of the total yield variance (49.7%), followed by variety (8.3%) and plant spacing configuration (1.8%). Sorghum growth duration was affected extremely significantly by ecological type, which contributed for 87.2% of its total variance. Plant height was mainly affected by genotype, which accounted for 81.6% of the total variance. All test varieties, whether they were bred in the south or the north, could reach maturity within 94 and 108 days, just before fall sowing in central China. Generally, sorghum growth duration becomes longer when a variety is introduced from south to north. A late-maturing variety, whether developed in the spring sowing and late-maturing regions, possibly could not reach maturity in the early-maturing region. The row spacing modes had no significant effect on sorghum yield, but the equal-row spacing mode consistently caused higher yields with only one exception; this might imply that equal-row spacing mode was more advantageous for boosting sorghum yield potential. In contrast, in-row plant spacing had a large influence on grain yield. Moreover, these results demonstrated that sorghum is a widely adapted crop and enables success in variety introduction across ecological zones.

## Data availability statement

The raw data supporting the conclusions of this article will be made available by the authors, without undue reservation.

## Author contributions

PY, data processing and statistical analysis and writing. Y-HS, K-YZ, FZ, Y-JT, X-NZ, NW, in charge of field experiments in each site. F-LK, F-JG, J-HL, J-XL, YG, WY, F-CG, D-DQ, ZW, G-XY participated in field experiments and data collection. F-XH, Z-YZ, G-YL, experiment design, analysis, writing, and revision. All authors contributed to the article and approved the submitted version.
