# Supplementary material for: Interaction of genotype-ecological type-plant spacing configuration in sorghum [Sorghum bicolor (L.) Moench] in China
Source: Front Plant Sci. 2023 Jan 12;13:1076854. doi: 10.3389/fpls.2022.1076854 (PMC9879661; doi:10.3389/fpls.2022.1076854)

Supplementary Figure S1 Configuration of the effect of six plant spacing pattern (row space mode and in-row plant space) on sorghum grain yield in four sites (JG, LS, HZ, and GX site) in 2020 and 2021. "*" indicates significant differences ($p<0.05$) within the group.

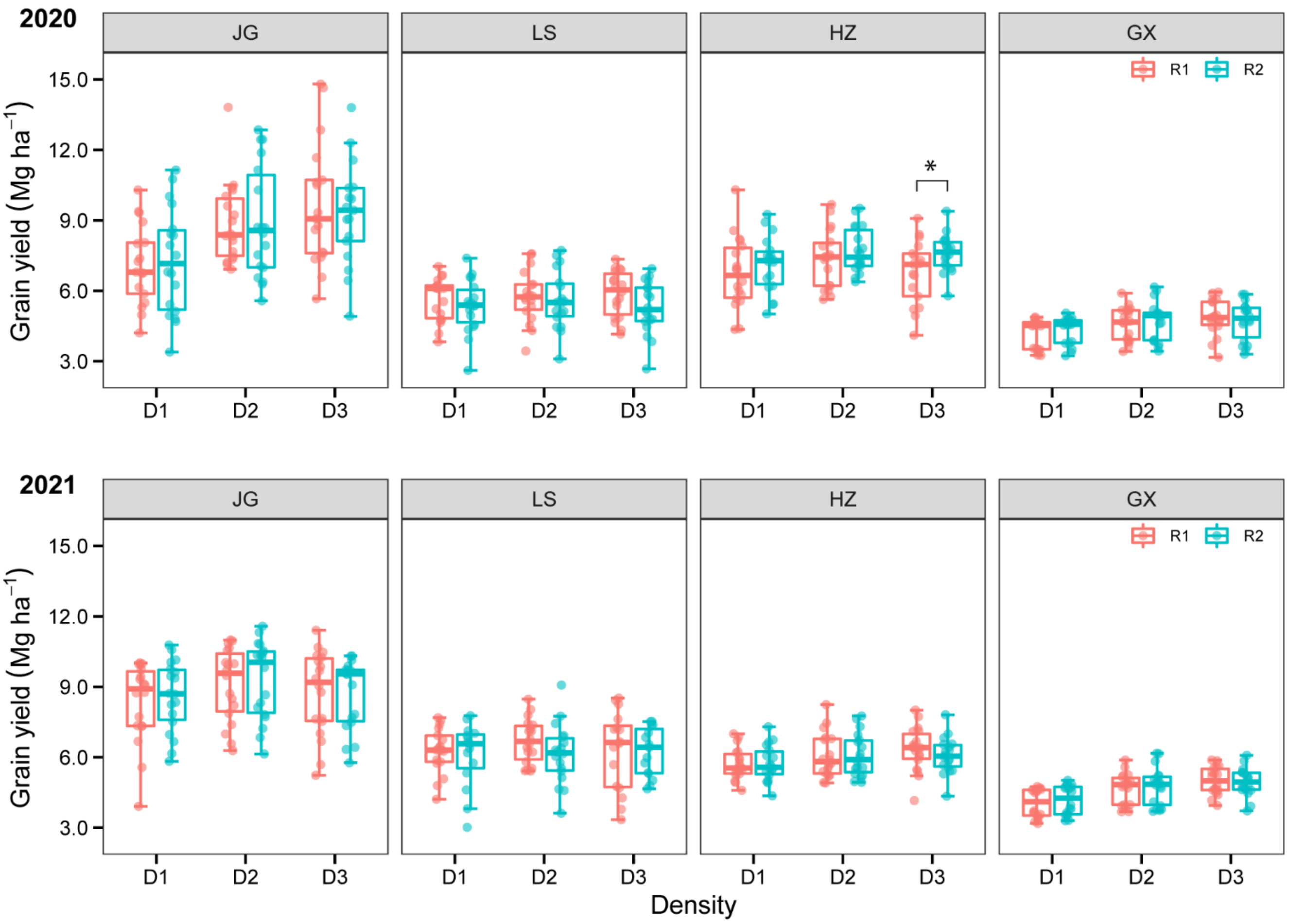

Supplement: Supplementary file 1 [file Image_1.pdf]
