# Supplementary material for: Interaction of genotype-ecological type-plant spacing configuration in sorghum [Sorghum bicolor (L.) Moench] in China
Source: Front Plant Sci. 2023 Jan 12;13:1076854. doi: 10.3389/fpls.2022.1076854 (PMC9879661; doi:10.3389/fpls.2022.1076854)
Supplement: Supplementary file 2 [file Table_1.docx]

**Supplementary Table S1** Topsoil characteristics of four sorghum experiment sites prior to planting in 2020 and 2021.

| Sites | pH | Organic matter | Total N | Available P | Available K |
| --- | --- | --- | --- | --- | --- |
|  |  | g kg^-1^ | g kg^-1^ | mg kg^-1^ | mg kg^-1^ |
| JG | 6.43 | 24.30 | 0.91 | 22.23 | 140.80 |
| LS | 6.83 | 32.37 | 0.87 | 47.93 | 145.70 |
| HZ | 8.44 | 15.12 | 0.77 | 96.89 | 322.80 |
| GX | 5.82 | 38.20 | 2.03 | 11.80 | 158.00 |

**Supplementary Table S2** Phenological phases of five sorghums (*Sorghum bicolor*, (L.) Moench.) varieties based on (Vanderlip and Reeves, 1972) at four experiment sites in 2020 and 2021.

| Year | Site | Cultivar | Sowing | Emergence | Flowering | Physiological maturity (PM) | Days from Sowing to PM |
| --- | --- | --- | --- | --- | --- | --- | --- |
|  |  |  | —————— (m-d) —————— | | | | (d) |
| 2020 | Gong-ZhuLing | A1 | 5-10 | 5-24 | 8-12 | 9-29 | 142 |
|  | (JG) | A2 | 5-10 | 5-24 | 8-1 | 9-22 | 135 |
|  |  | A3 | 5-10 | 5-24 | 8-4 | 9-24 | 137 |
|  |  | A4 | 5-10 | 5-24 | 8-6 | 9-25 | 138 |
|  |  | A5 | 5-10 | 5-24 | 7-31 | 9-26 | 139 |
|  |  | A6 | 5-10 | 5-23 | 7-31 | 9-24 | 137 |
|  | Shen-Yang | A1 | 5-8 | 5-17 | 8-1 | 9-9 | 124 |
|  | (LS) | A2 | 5-8 | 5-17 | 7-28 | 9-3 | 118 |
|  |  | A3 | 5-8 | 5-17 | 7-30 | 9-4 | 119 |
|  |  | A4 | 5-8 | 5-17 | 8-3 | 9-10 | 125 |
|  |  | A5 | 5-8 | 5-17 | 7-30 | 9-4 | 119 |
|  |  | A6 | 5-8 | 5-17 | 8-1 | 9-9 | 124 |
|  | Zheng-Zhou | A1 | 6-26 | 7-1 | 8-21 | 10-4 | 95 |
|  | (HZ) | A2 | 6-26 | 7-1 | 8-21 | 10-3 | 94 |
|  |  | A3 | 6-26 | 7-1 | 8-23 | 10-5 | 96 |
|  |  | A4 | 6-26 | 7-1 | 8-24 | 10-6 | 97 |
|  |  | A5 | 6-26 | 7-1 | 8-20 | 10-3 | 94 |
|  |  | A6 | 6-26 | 7-1 | 8-22 | 10-4 | 95 |
|  | Xing-Yi | A1 | 5-31 | 6-7 | 8-9 | 9-20 | 105 |
|  | (GX) | A2 | 5-31 | 6-7 | 8-9 | 9-20 | 105 |
|  |  | A3 | 5-31 | 6-7 | 8-13 | 9-24 | 109 |
|  |  | A4 | 5-31 | 6-7 | 8-13 | 9-24 | 109 |
|  |  | A5 | 5-31 | 6-7 | 8-9 | 9-18 | 103 |
|  |  | A6 | 5-31 | 6-7 | 8-9 | 9-18 | 103 |
| 2021 | Gong-ZhuLing | A1 | 5-15 | 5-22 | 8-6 | 9-24 | 125 |
|  | (JG) | A2 | 5-15 | 5-22 | 8-5 | 9-29 | 130 |
|  |  | A3 | 5-15 | 5-22 | 7-31 | 9-26 | 127 |
|  |  | A4 | 5-15 | 5-22 | 7-29 | 9-23 | 124 |
|  |  | A5 | 5-15 | 5-22 | 7-31 | 9-24 | 125 |
|  |  | A6 | 5-15 | 5-22 | 7-28 | 9-27 | 128 |
|  | Shen-Yang | A1 | 5-12 | 5-21 | 8-4 | 9-25 | 127 |
|  | (LS) | A2 | 5-12 | 5-21 | 7-23 | 9-20 | 122 |
|  |  | A3 | 5-12 | 5-21 | 7-23 | 9-20 | 122 |
|  |  | A4 | 5-12 | 5-21 | 7-23 | 9-18 | 120 |
|  |  | A5 | 5-12 | 5-21 | 7-23 | 9-12 | 114 |
|  |  | A6 | 5-12 | 5-21 | 7-23 | 9-20 | 122 |
|  | Zheng-Zhou | A1 | 6-14 | 6-18 | 8-17 | 9-30 | 104 |
|  | (HZ) | A2 | 6-14 | 6-18 | 8-17 | 10-1 | 105 |
|  |  | A3 | 6-14 | 6-18 | 8-23 | 10-4 | 108 |
|  |  | A4 | 6-14 | 6-18 | 8-20 | 10-2 | 106 |
|  |  | A5 | 6-14 | 6-18 | 8-17 | 9-29 | 103 |
|  |  | A6 | 6-14 | 6-18 | 8-18 | 10-2 | 106 |
|  | Xing-Yi | A1 | 6-2 | 6-9 | 8-6 | 9-20 | 103 |
|  | (GX) | A2 | 6-2 | 6-9 | 8-9 | 9-20 | 103 |
|  |  | A3 | 6-2 | 6-9 | 8-4 | 9-24 | 107 |
|  |  | A4 | 6-2 | 6-9 | 8-1 | 9-24 | 107 |
|  |  | A5 | 6-2 | 6-9 | 8-9 | 9-18 | 101 |
|  |  | A6 | 6-2 | 6-9 | 8-9 | 9-18 | 101 |

**Supplementary Table S3** Sorghum grain yield response to plant spacing and population as influenced by varieties at four experimental sites in 2020 and 2021

| Site | Cultivar | Plant spacing |  |  |  |  |  |  |
| --- | --- | --- | --- | --- | --- | --- | --- | --- |
|  |  | R1 (equal spacing pattern (60 cm)) | |  |  | R2 (wide-narrow spacing pattern (80/40cm) | | |
|  |  | Population change (x 1000 plants ha^-1^) | | |  |  |  |  |
|  |  | 111-83 | 166-83 | Mean |  | 111-83 | 166-83 | Mean |
|  |  | Δ Mg ha^-1^ | | | | | | |
| JG | A1 | 0.77 | 0.42 | 0.59 |  | 1.95 | 1.67 | 1.81 |
|  | A2 | 1.95 | 2.04 | 1.99 |  | 0.99 | 0.94 | 0.96 |
|  | A3 | 1.86 | 3.31 | 2.59 |  | 0.80 | 0.64 | 0.72 |
|  | A4 | 1.33 | 0.40 | 0.86 |  | 0.84 | 1.18 | 1.01 |
|  | A5 | 0.77 | 2.20 | 1.48 |  | 0.39 | 1.12 | 0.75 |
|  | A6 | 0.99 | 2.06 | 1.53 |  | 1.97 | 2.30 | 2.13 |
| LS | A1 | -0.37 | -0.43 | -0.40 |  | 1.02 | 0.21 | 0.62 |
|  | A2 | -0.04 | -0.04 | -0.04 |  | -0.30 | 0.67 | 0.18 |
|  | A3 | 0.91 | 0.53 | 0.72 |  | 0.67 | -0.42 | 0.13 |
|  | A4 | 0.98 | -0.05 | 0.47 |  | -0.77 | -1.51 | -1.14 |
|  | A5 | -0.29 | 0.23 | -0.03 |  | 1.02 | 0.63 | 0.82 |
|  | A6 | 0.46 | 0.22 | 0.34 |  | 0.19 | 0.36 | 0.27 |
| YX | A1 | 0.56 | 0.67 | 0.62 |  | 0.39 | 0.61 | 0.50 |
|  | A2 | 0.40 | 0.72 | 0.56 |  | 0.75 | 0.19 | 0.47 |
|  | A3 | 0.51 | 1.04 | 0.78 |  | -0.08 | 0.71 | 0.31 |
|  | A4 | 0.46 | 0.47 | 0.47 |  | 0.11 | 0.36 | 0.23 |
|  | A5 | 0.55 | 0.90 | 0.73 |  | 0.37 | 0.49 | 0.43 |
|  | A6 | 0.67 | 1.16 | 0.92 |  | 1.32 | 1.09 | 1.20 |
| HZ | A1 | -0.24 | 0.12 | -0.06 |  | 0.72 | 1.08 | 0.90 |
|  | A2 | 0.50 | 0.30 | 0.40 |  | 0.21 | -0.35 | -0.07 |
|  | A3 | 0.66 | 0.33 | 0.50 |  | 0.40 | 0.98 | 0.69 |
|  | A4 | 0.84 | 0.80 | 0.82 |  | 0.46 | -0.09 | 0.18 |
|  | A5 | 0.16 | 0.54 | 0.35 |  | 0.39 | 0.86 | 0.62 |
|  | A6 | 0.90 | 0.38 | 0.64 |  | 0.67 | -0.05 | 0.31 |
